# Telehealth Transition Assistance Program for Acute Spinal Cord Injury Caregivers: Protocol for a Mixed-Methods, Randomized Controlled Trial

**DOI:** 10.2196/28256

**Published:** 2021-03-29

**Authors:** Paul B Perrin, Scott D McDonald, Jack D Watson, Bradford S Pierce, Timothy R Elliott

**Affiliations:** 1 Virginia Commonwealth University Richmond, VA United States; 2 Central Virginia VA Health Care System Richmond, VA United States; 3 Texas A&M University College Station, TX United States

**Keywords:** spinal cord injury, telehealth, caregiver, methodology

## Abstract

**Background:**

While spinal cord injury (SCI) caregiving can be a rewarding experience, caregivers often experience reduced mental and physical health.

**Objective:**

This article describes the methodology of a study examining the efficacy of a newly developed telehealth Transition Assistance Program (TAP) for caregivers of individuals with acute SCI.

**Methods:**

A mixed-methods, randomized controlled trial is comparing TAP outcomes to that of a standard-of-care control. The study is recruiting for 48 months and incorporating quantitative outcome measures.

**Results:**

This study was funded by the Craig H. Neilsen Foundation in April 2017. It was approved by the institutional review boards at Virginia Commonwealth University and the Hunter Holmes McGuire Veterans Affairs Medical Center that same year. Participant recruitment and data collection began in 2018.

**Conclusions:**

This study is implementing and testing an SCI caregiver intervention unlike any created before, targeting a critical time period that, until now, other SCI caregiver interventions have overlooked. Research personnel intend to disseminate the intervention and study findings through the publication of manuscripts and presentations at conferences. If the current study shows improvements in caregiver or patient well-being, the TAP for SCI caregivers could become part of the standard of care for acute SCI.

**Trial Registration:**

ClinicalTrials.gov NCT03244098; https://www.clinicaltrials.gov/ct2/show/NCT03244098

**International Registered Report Identifier (IRRID):**

DERR1-10.2196/28256

## Introduction

Each year in the United States, 18,000 individuals experience a new spinal cord injury (SCI), and nearly 294,000 are living with SCI [1]. Individuals with SCI experience myriad medical complications [2] and reduced mental health [3,4], which bears on their own health-related quality of life (HRQoL) [5]. Although only one family member typically experiences an SCI, the injury affects the entire family [2,6]. Many family caregivers experience strain, depression, anxiety, lower general health [7,8], and burden [9-11].

Unfortunately, SCI caregivers perceive inadequate support from their social network, profound isolation, and insecurity in the weeks after hospital discharge [12,13]. The first few months are particularly stressful as caregivers cope with unstable social support and even their own wavering health [14]. Substantial research has been conducted on caregivers of individuals with various disabilities [15], but considerably less has focused on SCI caregivers. The current review uncovered several SCI caregiver interventions with empirical support; however, studies either did not target the specific acute discharge time period in the current study or psychosocial outcomes. Rodgers et al [16] developed the SCI Multiple-Family Group Treatment and targeted families who had been coping with SCI for 6 years on average. Schulz et al [11] compared the effectiveness of a caregiver-only intervention against an intervention for SCI caregiver-recipient dyads, but the individuals with SCI had sustained the SCI on average 7.7-9 years prior. Kurylo et al [17] developed the FOCUS program, which was incorporated by Elliott and Berry [18] into a formalized Problem-Solving Training program; however, no caregiver in either study participated during the first month after discharge. Molazem et al [19] showed positive results for a psychoeducational intervention on SCI caregiver HRQoL; however, the average length of time as a caregiver was 9 years. Hearn et al [20] showed that a web-based mindfulness intervention was helpful in reducing SCI caregiver anxiety and depression; however, the minimum length of time since injury was 1 year. Finally, Juguera Rodríguez et al [21] showed that simulation training for SCI caregivers during inpatient rehabilitation can improve competency in caregiver-related tasks but did not target psychosocial outcomes.

To improve SCI rehabilitation through stronger informal caregiving, this study is developing and evaluating a telehealth Transition Assistance Program (TAP) for caregivers of individuals with SCI during the transition from acute rehabilitation to home. This is the first SCI caregiver intervention to occur during this critical time and to target a host of important caregiving psychosocial variables. The TAP was previously developed for stroke caregivers [22] and is being modified for SCI and implemented at 2 state-of-the-art SCI rehabilitation facilities.

## Methods

### Study Design

This study is a prospective, mixed-method, randomized controlled trial incorporating a before-and-after design with a standard-of-care control wherein caregivers receive no formalized, structured postdischarge support. The study is recruiting over a period of 48 months.

### Setting

The TAP program is delivered via telehealth at a Veterans Affairs (VA) medical center and an academic medical center in the same urban area.

### Caregiver Guidebook Development

The research team developed a guidebook entitled “A Guidebook for SCI Caregivers” based heavily on research examining the needs of SCI caregivers, as well as on other published studies on SCI. Research has shown that the top reported needs of SCI caregivers include information, economic support, emotional support, community support, and respite needs [23]. This guidebook was created to target these needs directly and therefore includes chapters addressing (1) basic medical information about SCI; (2) common caregiver experiences; (3) SCI recovery issues such as disability, disruption in sense of self, social isolation, and depression; and (4) resources to assist SCI caregivers. An extensive formative evaluation of the guidebook was conducted including focus groups with SCI clinicians at the rehabilitation facilities at the VA and academic medical centers. The guidebook was piloted with SCI clinicians and caregivers who provided quantitative and qualitative feedback on the guidebook’s appropriateness.

### Participants, Recruitment, and Sample Size

The eligibility criteria for individuals with SCI and caregivers are the following: (1) between the ages of 18 and 89 years; (2) able to verbally communicate in English; (3) access to a computer, telephone, or other device capable of telecommunication; (4) no other serious mental or neurological disorders; (5) no active serious substance use disorder; and (6) being a dyad composed of 1 individual with a new diagnosis of SCI or significant new loss of function related to an old SCI and 1 individual identifying as a new informal caregiver. All individuals with SCI are those participating in a comprehensive residential rehabilitation program.

A power analysis was performed using G*Power 3.1. An estimated medium effect size of Cohen *f*=.25 was used to determine the sample size needed for a repeated-measures multivariate analysis of variance (RMANOVA) with 3 time points and 2 groups. With 80% power (1 - β), a sample size of 44 dyads is needed in order to detect the hypothesized medium-sized effects on the various outcomes. We selected a 48-month enrollment period because, based on SCI admissions data and previous studies conducted at the 2 rehabilitation sites, this window is necessary to enroll 44 patient-caregiver dyads (n=88), of which 22 dyads will be in the treatment group and 22 will be in the control group. This RMANOVA would uncover all large and medium-sized effects, but no small-sized effects.

Recruitment is conducted on site and in-person by either the research coordinator or the site’s principal investigator (PI). Patient-caregiver dyads are randomly assigned to the TAP treatment or control group, and each individual with SCI and caregiver is paid US $20 per data collection for a total of US $60 each.

### Randomization

A randomization schedule was created with a web-based, computerized random number generator. We maintain allocation concealment and eliminate possible selection or recruitment biases by keeping the randomization schedule concealed from on-site staff engaged in recruiting. A randomization schedule was generated by the PI who does not have any contact with participants, and a sealed envelope with the sequentially numbered randomization schedule was prepared prior to recruitment of any participants. After a project coordinator determines eligibility for a prospective dyad and obtains informed consent, the PI is notified and then opens the sealed envelope. The group assignment based on the predetermined sequence for that participant to 1 of the 2 groups is revealed at that point. If the participant is in the TAP group, an interventionist is then assigned to that participant. In order to control bias and preconceptions in collecting data, the interventionist does not collect baseline or follow-up data from participants with whom they intervene.

### Intervention Implementation

The TAP is carried out by a psychology PhD student interventionist who underwent rigorous training. The interventionist is responsible for scheduling and following up with caregivers under his or her care, and the same interventionist administers all sessions for a single caregiver in order to maintain consistency across sessions, unless extenuating circumstances arise. To ensure TAP intervention fidelity, the PI reviews the interventionists’ recordings for the first full 5 sessions that the interventionist conducts. A checklist was developed that covers the content specific to each session and an assessment of the interventionist’s interaction with the caregiver. The PI provides a summary of strengths, shortcomings, and recommendations for improvement so that any errors can be corrected immediately and not be repeated.

### Intervention

#### Session 1

The PI and collaborators trained a psychology PhD student to serve as an interventionist and provide the TAP. In preparation for Session 1 with the caregiver, the interventionist delivering the TAP meets with the facility’s rehabilitation team (and in particular, the SCI interdisciplinary team collaborators and consultants on the grant) to identify the primary difficulties anticipated for the individual with SCI after discharge. The interventionist takes notes on the particular needs and bring these notes to Session 1 with the caregiver. Before discharge, the interventionist implements Session 1, a 1-hour meeting with each caregiver in the intervention group only. The primary focus of Session 1 is to orient the caregiver to the TAP and prepare the caregiver for discharge home. The interventionist provides the caregiver the guidebook and orientation to it, encouraging the caregiver to use it as a resource (caregivers in the control group receive a copy of the caregiving guidebook after the final data collection). The interventionist asks what concerns the caregiver has caring for the individual with SCI after discharge, taking notes on the caregiver’s responses. The interventionist shares with the caregiver the difficulties that the rehabilitation team had anticipated the individual with SCI will experience after discharge. The interventionist provides support and helps the caregiver problem solve caregiving related to these issues.

#### Sessions 2-5

The interventionist administers four 1-hour telehealth clinic-to-home sessions with the SCI caregiver at 1, 2, 4, and 6 weeks after hospital discharge. One of the rehabilitation centers already had in place secure and encrypted telehealth technology that allows an interventionist on-site to meet virtually with a caregiver at home via personal computers or mobile devices. For caregivers who do not have a personal computer, mobile device, or adequate internet connection, a telephone-based approach is used. The individual with SCI may or may not be in the same room as the caregiver during these sessions, depending on the wishes of the caregiver. These sessions involve the same general format. The interventionist brings his or her notes from the previous sessions and from the rehabilitation team’s input. The interventionist reviews the content of these notes with the caregiver, checking in to see whether the problems are still present and to what extent. The interventionist engages in supportive problem solving and refers the caregiver to the guidebook sections relevant to the issues, walking the caregiver through those sections. Because of this format, the TAP is specifically designed for interventionists to tailor its use in future studies or administrations not only according to possible facility differences in what may be necessary for the intervention but also for differences in caregiver responsibilities and needs within a single facility. The entire structure of the TAP is centered around the caregiver’s most pressing needs. The interventionist takes notes on the continued problems and strategies for resolving them.

### Data Collection

#### Data Collection 1

After enrollment and immediately before discharge (as well as before Session 1 for the TAP group), demographic and baseline data are collected from the individual with SCI and caregiver separately by a study coordinator. A study coordinator reads items aloud from an assessment packet (unless a participant explicitly requests a paper-and-pencil format) to the individual with SCI and caregiver, noting participants’ responses. The caregiver packet includes validated measures of caregivers’ quality of informal care provided, depression, relationship satisfaction, burden, caregiving self-efficacy, health status, and positive affect/well-being. The packet for the individual with SCI includes validated measures of functional status, perceptions of quality of informal care received, depression, relationship satisfaction, self-perceived burden, health status, and positive affect/well-being. In rare cases, after Data Collection 1 has been conducted, where the patient’s discharge is moved to a later date, data collection may be readministered to ensure data accuracy.

#### Data Collection 2-3

**Figure 1:**
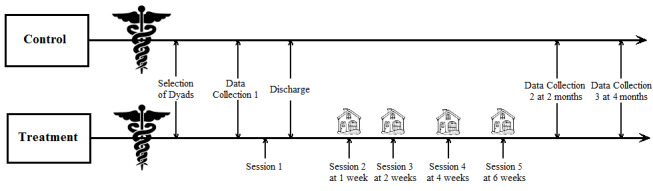
Randomized controlled trial timeline.

At 2 and 4 months, a study coordinator collects follow-up data from the individual with SCI and caregiver over the phone using the same validated measures as during the first data collection. A graphic depicting the study timeline can be seen in Figure 1.

### Outcome Measures

#### Exemplary Care Scale

The Exemplary Care Scale is administered for both patient and caregiver and is an 11-item self-report questionnaire that assesses the extent to which caregivers engage or do not engage in activities that help care recipients maintain dignity and respect [24]. Parallel caregiver and care recipient versions are available with equivalent measurement properties.

#### Spinal Cord Independence Measure Version III

The Spinal Cord Independence Measure Version III is completed by the patient and is a 19-item self-report questionnaire that assesses functioning of individuals with SCI in 3 domains: self-care, respiration and sphincter management, and mobility. Higher scores indicate greater patient functioning [25].

#### Center for Epidemiologic Studies Depression Scale-Revised

The Center for Epidemiologic Studies Depression Scale-Revised (CESD-R) is completed for both the patient and caregiver. The 20-item CESD-R [26] is the revised version of the original CESD [27], and higher total scores reflect higher depression symptomology.

#### Self-Perceived Burden Scale

The Self-Perceived Burden Scale is completed by the patient and assesses care recipients’ feelings of dependence and guilt regarding their caregiver’s difficulties [28]. The Self-Perceived Burden Scale contains 10 items, and higher scores indicate higher self-perception of being a burden.

#### Zarit Burden Interview

The Zarit Burden Interview [29] is completed by the caregiver and is a 22-item, self-report measure of caregiver burden with items referring to the caregiver and patient relationship and evaluating the caregiver’s health condition, psychological well-being, finances, and social life. Higher total scores indicate greater burden.

#### Revised Scale for Caregiving Self-Efficacy

The Revised Scale for Caregiving Self-Efficacy [30] is completed by the caregiver and measures 3 domains of caregiving self-efficacy: obtaining respite, responding to disruptive patient behaviors, and controlling upsetting thoughts.

#### 12-Item Short Form

Both the patient and caregiver complete the 12-item Short Form (SF-12). The SF-12 is one of the most widely used assessments of HRQoL in individuals with neurological conditions and their caregivers [31]. The SF-12 has 8 dimensions: physical function, role-physical, bodily pain, general health, energy/vitality, social function, role-emotional, and mental health. Higher scores indicate better HRQoL.

#### SCI-QOL Positive Affect and Well-Being Short Form

The Positive Affect and Well-Being Short Form [32] is completed by both the patient and caregiver and is a 10-item version of the National Institutes of Health Patient-Reported Outcomes Measurement Information System that has been tailored and optimized for the SCI population. Higher scores reflect greater positive affect and well-being.

### Data Analysis

A Greenhouse-Geisser–adjusted RMANOVA with 1 fixed effect of treatment condition (TAP vs control), the repeated measures over time (baseline and 2 and 4 months after discharge), and the treatment condition by time interaction will be conducted for the set of dependent variables. If an omnibus effect is found, follow-up Holm-Bonferroni–corrected ANOVAs will identify specific locations of effects. Our research has shown that these dependent variables rarely correlate with each other higher than .60 in SCI caregivers [5] so do not reach the .70 level that is often identified as problematic in RMANOVA [33].

## Results

This study was funded by the Craig H. Neilsen Foundation in April 2017 (Multimedia Appendix 1). It was approved by the institutional review boards at Virginia Commonwealth University and the Hunter Holmes McGuire Veterans Affairs Medical Center that same year. Participant recruitment and data collection began in 2018. As of March 2021, 31 caregiver-patient dyads (n=62) had enrolled in the study. Data analysis will begin toward the end of the grant cycle in March 2022, with results expected to be published in May 2022.

## Discussion

One primary goal of this study is to actively affect the care of individuals with SCI and their informal caregivers at a systematic level. As such, the caregiver guidebook developed for this study will be made freely available online so that any clinician may give it to any new, informal caregivers as they adjust to their new role. If the results are significant, the research team will make the intervention and results available through manuscripts and conference presentations. Detailed descriptions of the intervention will be published, and training videos will be made available online.

This study’s use of PhD psychology students as the study’s interventionists allows for a role that could create a future pipeline and documentation for training rehabilitation psychology students using the TAP system. Students could also receive course credit for practicum work in SCI rehabilitation settings while simultaneously gaining experience in telepsychology and with underserved populations. The use of PhD psychology students as interventionists helps defray health care costs and reduces the burden placed on the medical center’s staff [34].

The TAP intervention, especially if used as a training program for PhD students, could be implemented for minimal or even negligible health care costs. These savings could be passed to the individual with SCI and his or her informal caregiver. We also anticipate that participants will find the telepsychology intervention to be both more convenient and less of a strain on their resources than traditional, in-person sessions.

Given the study’s presence at both a VA medical center and an urban academic medical center, any study findings should have high generalizability to both the general population and veterans with SCI. Our TAP for SCI caregivers is innovative in that it fills one of the biggest gaps in SCI rehabilitation by aiming to improve the mental health of and quality of informal care provided by caregivers immediately as they transition into their caregiving role. The study’s telehealth format surmounts geographical barriers and links SCI caregivers via telehealth technology to the specialized center from which the individual with SCI received acute rehabilitation, thereby overcoming the discontinuity in care that all too often affects individuals with SCI and their family after discharge. If shown in the proposed study to improve caregiver mental health, informal care, and SCI rehabilitation, the TAP for SCI caregivers could be exported and evaluated much more widely across other rehabilitation facilities in the United States and hopefully become part of the standard of care for SCI.

## Appendix

Multimedia Appendix 1 Peer-review report by Craig H Neilsen Foundation.

## Abbreviations

CESD-R: Center for Epidemiologic Studies Depression Scale-Revised
HRQoL: health-related quality of life
PI: principal investigator
RMANOVA: repeated-measures multivariate analysis of variance
SCI: spinal cord injury
SF-12: 12-item Short Form
TAP: Transition Assistance Program
VA: Veterans Affairs
