# Supplementary material for: Telehealth Transition Assistance Program for Acute Spinal Cord Injury Caregivers: Protocol for a Mixed-Methods, Randomized Controlled Trial
Source: JMIR Res Protoc. 2021 Mar 29;10(3):e28256. doi: 10.2196/28256 (PMC8086783; doi:10.2196/28256)
Supplement: Multimedia Appendix 1 [file resprot_v10i3e28256_app1.pdf]

CRAIG·H NEILSEN FOUNDATION

proposalCENTRAL

Proposal Information

Applicant: Perrin, Paul
Title Randomized Clinical Trial of a Telehealth Transition Assistance Program for SCI Caregivers
Type/Competition PSR Studies and Demonstration Projects
Institution Virginia Commonwealth University
App # 439128

Committee: PSR 2017 FGA

Summary Statement

Text Admin Summary:
The Neilsen Foundation has conditionally approved funding of this proposal. Please see the reviewer summaries for a discussion of strengths and weaknesses. The following issue(s) deserve particular attention: The study of caregiving during the specific timeframe when an individual with SCI is transitioning from hospital to home is an important aspect of the proposal. This unique focus of the well-designed study, along with the strong research team and setting, led to high enthusiasm among reviewers. The PI's integration of the recommended administrative follow-up (to update measures used, add caregiver input in developing materials and revise the proposed recruitment plan) will further strengthen the project.

Reviewer Role: Primary (237820)

OVERALL IMPACT
After considering all of the review criteria, summarize the significant strengths and weaknesses of the application. In this funding category, does this project address important gaps or propose cutting-edge ideas, interventions and/or test approaches that have great potential to have a positive impact for those living with SCI? Please state the likelihood that the project scope suits this funding level and will develop an area of work that has the potential to exert a sustained powerful influence on the SCI field and/or be an important contributor to the field of SCI research.

:
Strengths of this project include an outstanding team of investigators with vast experience in caregiver interventions, previous experience with telehealth modalities and a sound research design. The rationale for the study is well supported by the existing literature and addresses a significant gap in caregiver support - the transition from rehab to home. Few and relatively insignificant weaknesses include some of the outcome measures selected and a possibly inadequate sample - over-recruitment is probably warranted to ensure complete data on 44 dyads. This project, if successful will lead to seeking further funding to expand this program to other sites and develop a standardized rehabilitation protocol utilizing the TAP program.

SIGNIFICANCE
1. Does the project address an important problem or a critical barrier in the field?

2. If the Aims of the project are achieved, how would this work change or enhance current methods, technologies, treatments, services, or interventions?

:
Caregivers are often thrown into "real life" with little training and have to face difficulties at home that may not have been anticipated during rehabilitation. Preparing caregivers with as many skills as possible prior to return home with their family members is critical to the overall well-being and health of both the caregiver and care recipient. This study addresses a gap that currently exists and addresses the transition to home using a telehealth Transition Assistance Program (TAP) to determine if such an intervention can minimize depression, improve relationship satisfaction, self-efficacy, health status and well being among informal caregivers. Existing programs for people with stroke have been relatively effective and the investigators (who conducted the stroke study) are modifying this intervention to caregivers of people with SCI. The features of this program, if successful, could be implemented relatively easily and at low cost in other settings and could lead to better caregiver and care recipient outcomes for those engaging in TAP.

RELEVANCE
1. How is this project relevant to the mission of the Neilsen Foundation?

2. How is this project relevant to the goal of the Psychosocial Research portfolio?

:
The project is highly relevant to the CHNF mission to improve quality of life for people with SCI and with the PSR portfolio goals in that it explores a previously tested (and to be modified) intervention to assist caregivers in better managing their caregiving role and responsibilities. Better caregiver outcomes have been demonstrated to relate to better care recipient outcomes.

INVESTIGATOR(S)
1. Are the PI, collaborators, and other contributors well suited to the project?

2. If the PI is a junior investigator, does he/she have appropriate experience, training and facilities to do the proposed work? If the PI is an established investigator, has he/she demonstrated an ongoing record of accomplishments that have advanced their field(s)?

:
The team of investigators is extremely well suited to carry out this project. Dr. Perrin, although relatively early in his career, has focused on developing caregiver interventions. Dr. Elliott is one of the top names in caregiver research in SCI and has been instrumental in developing cognitive behavioral interventions for caregivers. He has published widely in this area. Dr. McDonald brings the focus to veterans and although most of his work has related to brain injury ,he will provide useful collaboration from the VA. Dr. Pickett has utilized telehealth methodology in his career with other disability populations. In all, this team is excellent in composition to carry out the specific intervention, analysis and dissemination of the study findings.

INNOVATION
1. Does the project challenge and seek to shift current research or clinical practice/program intervention paradigms by utilizing novel theoretical concepts, approaches or methodologies, instrumentation, or interventions?

2. And/or does this application apply concepts, approaches or methodologies, instrumentation, or interventions from another field of research to spinal cord injury?

3. And/or is a refinement, improvement, or new application of theoretical concepts, approaches or methodologies, instrumentation, or interventions proposed?

:
The proposed TAP project is innovative, not in the methodology itself, but in the timing of the intervention. By addressing issues with caregivers during rehab and as they transition home. Other identified interventions have not been targeting this critical time period. The use of the telehealth component after caregivers have left the rehab setting eliminates geographical barriers and provides continuity of intervention with the same therapist conducting the follow-up. The investigators did an excellent job of homework by identifying existing funded caregiver projects from the CHNF and highlighting that although they each have potential benefits, none address the critical transition period that the current study addresses. The experience of the investigators in delivering interventions to caregivers strengthens the project considerably, and the fact that this intervention has been used in other populations is promising.

APPROACH
1. Are the overall strategy, methodology, and analyses well-reasoned and appropriate to accomplish the specific aims of the project, within the proposed project period and by the project team described? Are potential problems, alternative strategies, and benchmarks for success presented?

2. Are preliminary data provided to support the feasibility of the project? Or, if the project is in the early stages of development, will the strategy establish feasibility and will particularly risky aspects be managed adequately?

3. If the project involves clinical and/or community-based research: A) are the plans for protection of human subjects from research risks described and adequate; and B) are the plans for recruitment of patients/participants appropriate?

:
Many of the citations supporting the project are rather dated (other than those from the investigators themselves). Nonetheless, this is an important topic and the approach to study the issue is appropriate and well designed. Strengths are having strong preliminary data from the stroke study and the ability to modify that program reduces start-up and operational costs. Of some concern is that the developed guidebook will be evaluated by clinicians - but it seems that evaluation by caregivers themselves would be critical. Not sure why FIM is being used - it is problematic in general and while widely used and validated in SCI (and for telephone administration) it may not be the best tool - consider SCIM? The ECS rating of quality of care has only been used in older adults and while it is suggested that it is applicable to other populations, this has not been validated. Is the walk/wheelchair version of the SF-12 being used? It is more relevant to this population. The recruitment and enrollment plan does not seem to account for attrition. Even though the intervention is relatively short, there is likely to be drop-out, particularly in the control group. Human subjects protections are well described and appropriate. The table outlining challenges and how they will be addressed is excellent.

ENVIRONMENT
1. Will the institutional environment in which the work will be done contribute to the probability of success?

2. Are the institutional support, physical equipment and other resources available to the investigators adequate for the project proposed?

3. Will the project benefit from unique features of their environment, subject populations, or collaborative arrangements?

:
The environment is entirely appropriate for the study activities. There is support from the VA to use space for the study. Telehealth capabilities are in place.

NON-SCORED CRITERIA
(PLEASE NOTE THAT THE SCORE FOR THIS NON-SCORED SECTION MUST BE A "50-NONSCORED." PLEASE DISREGARD SCORING OPTIONS 1-9 FOR THIS SECTION ONLY.)

Please provide any important or relevant comments on each of the non-scored criterion below.

1. Budget

2. Ethics/Safety

3. Other/Additional Comments for the Applicant

4. RESUBMISSION: When reviewing a Resubmission, the committee will evaluate the application as now presented, taking into consideration the responses to comments from the previous scientific review group and changes made to the project.

:
No concerns

Reviewer Role: Secondary (237821)

OVERALL IMPACT
After considering all of the review criteria, summarize the significant strengths and weaknesses of the application. In this funding category, does this project address important gaps or propose cutting-edge ideas, interventions and/or test approaches that have great potential to have a positive impact for those living with SCI? Please state the likelihood that the project scope suits this funding level and will develop an area of work that has the potential to exert a sustained powerful influence on the SCI field and/or be an important contributor to the field of SCI research.

:
The proposed study addresses an important problem, caregiver burden and distress by modifying an intervention that was successful with stroke caregivers and applying it to SCI caregivers. The applicant anticipates potential problems and lists strategies to mitigate study risk. The randomized controlled design is a strength; the telehealth delivery is apt to be cost effective and reproducible. The project scope suits this funding level and is highly likely to develop an area of work that has the potential to exert a sustained powerful influence on the SCI field.

SIGNIFICANCE
1. Does the project address an important problem or a critical barrier in the field?

2. If the Aims of the project are achieved, how would this work change or enhance current methods, technologies, treatments, services, or interventions?

:
This application addresses an important problem in SCI rehabilitation, caregiver distress. Many SCI and caregivers experience long-term distress related to post-SCI functional changes that makes post-SCI social and community reintegration challenging. Many caregivers are significantly distressed during the first year of caregiving for a person with SCI. They experience strain, depression, and anxiety, as well as limited time for rest and lower general health. They often feel hopeless and trapped within their circumstances. Often SCI caregivers experience inadequate support and response to their needs from others in their social network during the transition home. Given these needs, little research on interventions for SCI caregivers has been reported. If the Aims of the project are achieved, development and initial evaluation of caregiver intervention would lead to enhanced interventions for caregivers.

RELEVANCE
1. How is this project relevant to the mission of the Neilsen Foundation?

2. How is this project relevant to the goal of the Psychosocial Research portfolio?

:
The proposal addresses the Craig H. Neilsen Foundation's goal to support research on the psychological and social factors that affect the quality of life of people affected by spinal cord injury. Informal caregiving relationships are critical to quality of life following SCI.

INVESTIGATOR(S)
1. Are the PI, collaborators, and other contributors well suited to the project?

2. If the PI is a junior investigator, does he/she have appropriate experience, training and facilities to do the proposed work? If the PI is an established investigator, has he/she demonstrated an ongoing record of accomplishments that have advanced their field(s)?

:
The PI, collaborators, and other contributors are well suited to the project. Dr. Perrin is a tenured associate professor and a licensed clinical psychologist; he has served as PI of an NIH-funded, multi-site randomized clinical trial of stroke caregiver interventions, 125 peer-reviewed publications, and extensive clinical expertise in SCI rehabilitation. Dr. Elliott has extensive research experience focused on the psychosocial aspects of SCI and developing and testing SCI caregiver interventions. Dr. McDonald is an SCI psychologist at the Richmond VA and has published extensively on psychosocial adjustment following SCI. Dr. Pickett is the Associate Chief and Supervisory Clinical Psychologist, Mental Health. Collectively, they bring the requisite skills and experience to conduct this study successfully.

INNOVATION
1. Does the project challenge and seek to shift current research or clinical practice/program intervention paradigms by utilizing novel theoretical concepts, approaches or methodologies, instrumentation, or interventions?

2. And/or does this application apply concepts, approaches or methodologies, instrumentation, or interventions from another field of research to spinal cord injury?

3. And/or is a refinement, improvement, or new application of theoretical concepts, approaches or methodologies, instrumentation, or interventions proposed?

:
This application applies approaches and methodologies, and interventions from stroke caregiver interventions to spinal cord injury. The intervention is innovative by addressing a significant gap in SCI rehabilitation by addressing the mental health of and quality of informal care provided by caregivers. The telehealth format reduces geographical barriers and connects SCI caregivers via telehealth technology to the rehabilitation center where the individual with SCI received acute rehabilitation, thus providing continuity in care. This intervention for SCI caregivers could be adopted and evaluated at other rehabilitation facilities perhaps become a standard of care for SCI.

APPROACH
1. Are the overall strategy, methodology, and analyses well-reasoned and appropriate to accomplish the specific aims of the project, within the proposed project period and by the project team described? Are potential problems, alternative strategies, and benchmarks for success presented?

2. Are preliminary data provided to support the feasibility of the project? Or, if the project is in the early stages of development, will the strategy establish feasibility and will particularly risky aspects be managed adequately?

3. If the project involves clinical and/or community-based research: A) are the plans for protection of human subjects from research risks described and adequate; and B) are the plans for recruitment of patients/participants appropriate?

:
The overall strategy, methodology, and analyses are well-reasoned and appropriate to accomplish the specific aims of the project, within the proposed project period and by the project team described. The proposed randomized controlled trial will modify and evaluate a telehealth Transition Assistance Program (TAP) for informal caregivers of individuals with SCI during the transition from hospital to home. The program was developed for stroke caregivers and leads to decreased caregiver strain and depression. The applicant reports preliminary data that support the feasibility of the project. The investigators plan to modify the Program for SCI caregivers and implement it at a SCI rehabilitation facility where telehealth technology is already in place. The study aims are reasonable and logical, to: 1. Evaluate the program's effects on caregivers' quality of informal care provided, depression, relationship satisfaction, burden, caregiving self-efficacy, health status, and positive affect/well-being; 2. Evaluate the Program's effects on on patients' functional status, perceptions of quality of informal care, depression, relationship satisfaction, self-perceived burden, health status, and well-being; 3. Identify barriers and facilitators to implementing the TAP for SCI caregivers; and 4. Compare the health care cost and utilization experiences of the control group to the intervention group. The population from which a sample of 44 patient-caregiver dyads is sufficiently large. The applicant gives unusually thoughtful consideration and discussion of potential problems and alternatives, enhancing confidence that the trial will be completed successfully. The PI will be on-call to consult regarding any emergencies or adverse events. Plans for protection of human subjects from research risks appear to be adequate, and plans for recruitment of caregivers and patients appears to be appropriate?

ENVIRONMENT
1. Will the institutional environment in which the work will be done contribute to the probability of success?

2. Are the institutional support, physical equipment and other resources available to the investigators adequate for the project proposed?

3. Will the project benefit from unique features of their environment, subject populations, or collaborative arrangements?

:
The institutional environment in which the work will be done is likely to contribute to the probability of success. The institutional support, physical equipment and other resources available to the investigators appear to be adequate for the project proposed. This proposal benefits from the expertise, resources, and environment of the research team at VCU, the Richmond VA, and Texas A&M University.

NON-SCORED CRITERIA
(PLEASE NOTE THAT THE SCORE FOR THIS NON-SCORED SECTION MUST BE A "50-NONSCORED." PLEASE DISREGARD SCORING OPTIONS 1-9 FOR THIS SECTION ONLY.)

Please provide any important or relevant comments on each of the non-scored criterion below.

1. Budget

2. Ethics/Safety

3. Other/Additional Comments for the Applicant

4. RESUBMISSION: When reviewing a Resubmission, the committee will evaluate the application as now presented, taking into consideration the responses to comments from the previous scientific review group and changes made to the project.

:
No response entered

©2007-2016 Altum, Inc. All rights reserved Contact Us Terms of Service Acceptable Use Policy Privacy Policy
